# CD4 and Viral Load Dynamics in Antiretroviral-Naïve HIV-Infected Adults from Soweto, South Africa: A Prospective Cohort

**DOI:** 10.1371/journal.pone.0096369

**Published:** 2014-05-15

**Authors:** Neil A. Martinson, Nikhil Gupte, Reginah Msandiwa, Lawrence H. Moulton, Grace L. Barnes, Malathi Ram, Glenda Gray, Chris Hoffmann, Richard E. Chaisson

**Affiliations:** 1 Perinatal HIV Research Unit, Faculty of Health Sciences, University of the Witwatersrand, Johannesburg, South Africa; 2 School of Medicine, Johns Hopkins University, Baltimore, Maryland, United States of America; 3 Bloomberg School of Public Health, Johns Hopkins University, Baltimore, Maryland, United States of America; 4 Department of Science and Technology/National Research Foundation Centre of Excellence for Biomedical TB Research, Johannesburg, South Africa; University of Southampton, United Kingdom

## Abstract

**Background:**

CD4 count is a proxy for the extent of immune deficiency and declines in CD4 count are a measure of disease progression. Decline in CD4 count is an important component: for estimating benefits of ARV treatment; for individual level counselling on the rapidity of untreated disease progression and prognosis; and can be used in planning demand for health services. Our objective is to report CD4 decline and changes in viral load (VL) in a group of HIV-infected adults enrolled in a randomized trial of preventive treatment for TB in South Africa where clade C infection predominates.

**Methods:**

HIV-infected, tuberculin skin test positive adults who were not eligible for antiretroviral (ARV) treatment were randomized to a trial of preventive treatment from 2003–2005. VL and CD4 count were assessed at enrollment and CD4 counts repeated at least annually. During follow-up, individuals whose CD4 counts decreased to <200 cells/mm^3^ were referred for antiretroviral therapy (ART) and were analytically censored.

**Results:**

1106 ARV naïve adults were enrolled. Their median age was 30 years and male to female ratio was 1∶5. Median baseline CD4 count was 490 cells/mm^3^ (IQR 351–675). The overall mean decline in CD4 count was 61 cells/mm^3^ per annum. Adjusting for age, gender, baseline hemoglobin, smoking and alcohol use had little impact on the estimate of CD4 decline. However, VL at baseline had a major impact on CD4 decline. The percent decline in CD4 count was 13.3% (95% CI 12.0%, 14.7%), 10.6% (95% CI 8.8%, 12.4%), and 13.8% (95% CI 12.1%, 15.5%) per annum for baseline VLs of <10,000 (N = 314), 10,001–100,000 (N = 338), >100,000 (N = 122) copies/ml.

**Conclusions:**

Our data suggests that six and a half years will elapse for an individual's CD4 count to decline from 750 to 350 cells/mm^3^ in the absence of ART.

## Introduction

Since Mellor's et al landmark papers on the relative prognostic value of viral load on CD4 count decline [1], [2], there have been few reports describing CD4 cell decline and the influence of HIV RNA on CD4 decline – particularly from developing settings where the HIV subtypes, host factors, and route of HIV acquisition differ to those in North America. Yet infecting HIV type and subtype appear to have a major impact on the rate of CD4 decline [3]–[5]. Estimates of CD4 count decline, however, are important to predict the time to CD4 count based antiretroviral therapy (ART) initiation thresholds. For populations, accurate characterization of CD4 decline is valuable for: epidemiological modeling; forecasting resource needs; and estimating cost-benefits of HIV prevention including initiation of ART at higher CD4 counts. Reports of CD4 decline from South Africa, where HIV-1 subtype C is the predominant infecting strain, have described CD4 decline in ART naïve patients but lack contemporaneous HIV viral load data [6]–[8].

The overall aim was to describe CD4 and viral load dynamics in a closely monitored group of HIV-infected antiretroviral therapy naïve adults, in South Africa, who entered the study with CD4 counts either above or close to 500 cells/mm^3^ and were followed-up at semi-annual scheduled visits for almost four years. The aims for this paper were achieved.

## Methodology

### Ethics Statement

Approval was obtained from the Institutional Review Boards of Johns Hopkins University and the University of the Witwatersrand. Written informed consent was obtained from all participants.

### Participants and Method

We conducted a secondary analysis of a cohort of tuberculin skin test (TST) positive (≥5 mm), HIV-infected adults, recruited from September 2003 to June 2005 to a randomized trial of preventive treatment against TB. The trial compared three novel TB preventive treatment regimens with the World Health Organization's recommended standard – isoniazid for six months [9]. Recruitment and follow-up of this cohort have been previously described [10]. Participants were HIV-infected adults, at least 18 years of age who did not have active TB, which was excluded by symptom screen, chest X-ray, and clinical examination with appropriate laboratory investigations. In addition participants were excluded if they were candidates for ART (at the then CD4 count initiation threshold of 200 cells/mm^3^) or had previously received more than 2 months of treatment for TB disease. A baseline CD4 count and viral load were taken either during the screening process or within 90 days after randomization. CD4 counts were measured annually until 2004 when measurement was increased to either six-monthly or, If a prior CD4 count was <350 cells/mm^3^, CD4 counts were enumerated three-monthly to ensure timely referral for antiretroviral therapy. HIV RNA was assayed annually. A physician evaluated all participants annually and when clinically indicated.

We included follow-up time from the date of first CD4 count to the earlier of the date of ART initiation or termination from the study. In addition, to be absolutely sure that no participants who were taking ART were included in this analysis, we censored follow-up time of those participants who, despite not self-reporting ART, had an HIV RNA <400 copies/mL after having HIV RNA levels >1000 copies/mL at all previous visits.

### Statistical Methods

Baseline characteristics, which included socio-demographic, clinical and laboratory measures, were summarized using frequency and percentages for categorical variables and by medians with Inter-quartile ranges (IQR) for continuous variables. We report the CD4 decline over time both in absolute and relative terms: cells/mm^3^ per month and %, respectively, using a random effects model [11]. We also investigated how declines varied by baseline viral load, which was log_10_ transformed and stratified into mutually exclusive categories reflecting prior published work: <500 c/mL, 501–3000 c/mL, 3001–10,000 c/mL, 10,001–30,000 c/mL, and >30,000 c/mL. We used a mixed linear model to calculate CD4 change, over time, incorporating random effects to account for multiple updated measures. We identified individual factors that impacted on CD4 decline by building a model with an interaction between time and that factor. When the interaction was plausible and statistically significant (at α = 0.05) we included it in multivariable analysis. We also assessed significance of factors as intercepts in the mixed linear model. To allow direct comparison of our data with that of Mellors et al, we plotted CD4 declines, categorized by the baseline viral load categories used in their analysis. In addition to assessing how CD4 declines varied by viral load, the log_10_ viral load slope within each CD4 count category was also estimated using a random effects model adjusted for baseline variables.

## Results

This analysis included 1106 ARV naïve adult participants –184 (17%) were men;– representing 96.3% of the total randomization cohort of the parent trial (Table 1). Participants in this analysis were followed up for a median of 3.7 years (IQR: 2.4–4.5) prior to initiating antiretroviral therapy or being terminated from the study; 166 started antiretroviral therapy at a median CD4 count of 192 cells/mm^3^ (IQR 149–243) and were censored at the time of antiretroviral initiation. Another 42 participants were also censored, despite not reporting ART initiation, as their HIV RNA values reduced from a prior level of >1000 copies/ml to <400 copies/mL. Sixty-four (6%) died whilst being followed up. The remaining cohort contributed a total of 5,960 CD4 counts and 2,569 viral load measurements. Median CD4 count and viral load at enrollment was 490 cells/mm^3^ (IQR 351–675) and 16,050 copies/ml (IQR 3,680–57,400), respectively. At termination or censoring, median CD4 count and viral load were 345 cells/mm^3^ (IQR 220–523) and 7,567 copies/mL (IQR 2,036–24,423), respectively.

**Table 1:** Baseline characteristics of the cohort of the sub-group of ARV naïve participants followed up in a randomized trial of preventive treatment against TB.

Characteristics	N = 1106 n (%) or Median (IQR)
Male Gender (%)	184 (17%)
Median Age (Years)	28 (23–34)
Median Years of Education)	2 (2 −3)
Height (m)	1.60 (1.56–1.65)
Weight (Kg.)	64.7 (57.2–75.2)
BMI (Kg/m^2^)	24.8 (22.1–28.9)
Baseline CD4	490 (351–675)
>500	532 (48%)
350–500	314 (28%)
200–350	260 (24%)
Baseline VL	16050 (3680–57400)
Baseline log10 VL	4.2 (3.6–4.8)
<5000	299 (27%)
5,000–100,000	569 (51%)
>100,000	238 (22%)
Median time in Follow-up (months)	44 (29–54)

### CD4 Decline

**Figure 1:**
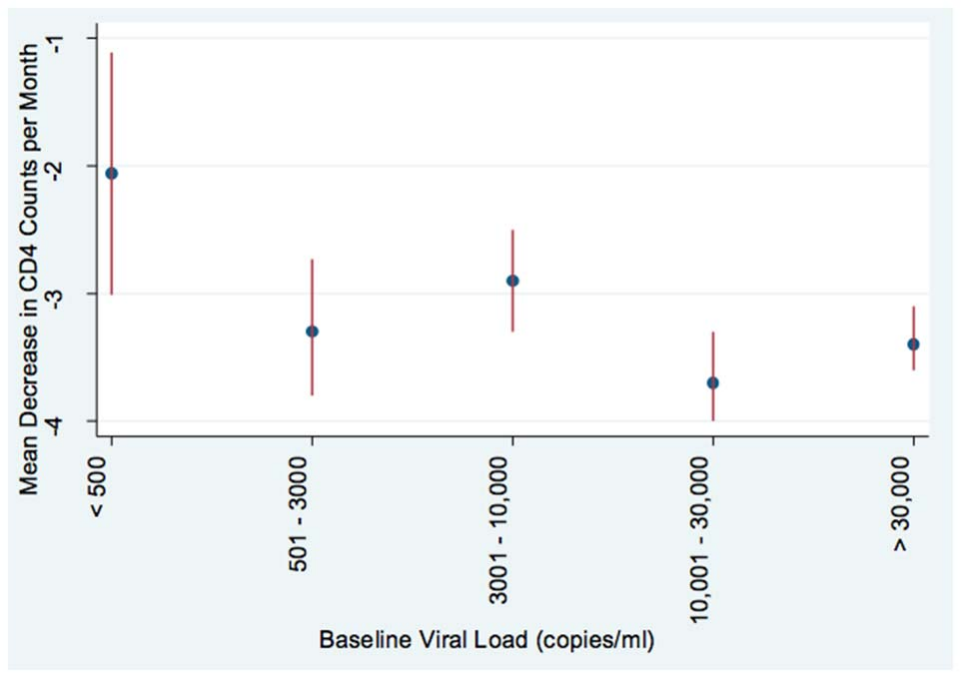
Mean CD4 count decline (cells/mm^3^ per month) with 95% confidence interval bars stratified by baseline viral load.

The overall unadjusted CD4 count decline (from baseline) was 3.2 cells/mm^3^ per month (95% CI 3.0–3.4). Absolute and proportional declines in CD4 counts by baseline CD4 strata are shown in table 2. The overall average CD4 count decline after adjustment for gender, age, education level and HIV RNA using the random effects model, was 3.3 cells/mm^3^ per month (95% CI: 3.0%–3.5%) (Table 3). Of the factors included in this multivariable model, viral load had the greatest impact on CD4 decline; for each unit increase in log_10_ baseline viral load, there was an increase in average CD4 count decline of 135 cells/mm^3^ per month. CD4 decline by viral load strata is shown in Figure 1. Men had lower CD4 count declines compared to women; unadjusted CD4 count decline in 184 men with 594 person years of follow-up was 2.6 cells/mm^3^ per month (95% CI 3.04–3.20) whereas CD4 decline in 921 women with 3,109 person years of follow-was 3.3 cells/mm^3^ per month (95% CI: 3.12–3.52) (p = 0.014); and after adjustment, was 2.8 cells/mm^3^ per month (2.3–3.3) and 3.4 cells/mm^3^ per month (3.1–3.6), in men and women, respectively (p = 0.057).

**Table 2:** Estimated mean CD4 decline per month by baseline CD4 category, using the random effects model adjusting for baseline socio-demographic characteristics.

Baseline CD4 Category (cells/mm^3^)	N	Mean CD4 Decline		p-value*
		Absolute (cells/mm^3^) per month (95%CI)	Proportional (%) decline per month (95%CI)	
200–350	260	1.43	0.50	0.13
		(1.08–1.78)	(0.37–0.63)	
350–500	314	2.27	0.54	0.41
		(1.99–2.54)	(0.48–0.61)	
500–1000	465	4.04	0.61	0.56
		(3.74–4.33)	(0.57–0.66)	
≥1000	67	5.30	0.43	0.12
		(3.78, 6.83)	(0.30–0.56)	

*The p-value tests if the change in CD4 count differs from 0.

**Table 3:** Estimated mean CD4 decline using the random effects model; univariable and multivariable analysis.

Characteristics	Univariate Analysis		Multivariate Analysis	
	Mean change in CD4 Counts (cells/mm^3^ per month)	p-value	Mean change in CD4 Counts (cells/mm^3^ per month)	p-value
Time (Months)*	−3.2	P<0.001	−3.3	p<0.001
	(−3.4, −3.0)		(−3.5, −3.0)	
Log 10 Viral Load	−132	P<0.001	−135	P<0.001
	(−146, −119)		(−150, −121)	
Age (Years)	−1.2	P = 0.13	−0.01	0.03
	(−2.7, 0.33)		(−0.009, −0.0004)	
Male Gender	−38.9	0.04	−46.6	0.014
	(−75.2, −2.6)		(−0.83.8, −9.41, 86.1)	
Highest school grade	20.2	0.07	−0.39	0.97
	(−1.98, 42.4)		(−22.1, 21.3)	
Weight (kg)	2.1	P<0.001		
	(1.1, 3.0)			
BMI (kg/m^2^)	5.8	p<0.001	4.5	0.001
	(3.1, 8.5)		(1.88, 7.1)	
Active Tuberculosis	−136	P<0.001	−56.4	0.049
	(−193.5, −79.1)		(−112.4, −0.26)	

*Variable ‘time in months’ is measuring CD4 change over time and the p-value tests if the change differs from 0.

### Viral Load Changes

Using random effects modeling, the annual slope viral load was 0.03 log_10_ copies/ml, but was not significantly different to zero. The effect on the slope of log_10_ viral load by baseline CD4 counts was assessed in the four baseline CD4 categories: 200–350, 350–500, 500–1000 & >1000. In each of these CD4 categories, the annual change in log_10_ viral load did not differ significantly from zero, even after adjusting for a variety of variables.

### Long-Term Non-Progressors and Elite Controllers

Of all 1,106 participants, 142 had follow-up of at least five years. Of these, 98/142 (69%) had a baseline CD4 count of at least 500 cells/mm^3^ of whom 29/98 (30%) or 2.6% of the entire cohort maintained their CD4 counts ≥500 cells/mm^3^. The median baseline HIV RNA of these 29 long term non-progressors was 3,410 copies/ml (IQR: 443–7,600) whereas the median baseline viral load in those whose CD4 count declined to below 500 cells/mm^3^ at any time in follow-up was 12,300 copies/ml (IQR: 6,540–27,200) (p<0.0001). Of the entire group of 1,106 adults, 70 (6.3%) had baseline viral load of less than 400 copies/mL of whom 35/70 (50%) or 3.2% of the entire cohort persistently maintained their viral load below 400 copies/ml for their entire follow-up. The other 35 participants with undetectable viral load at baseline and who had a subsequent detectable viral load, had a median first detectable viral load of 1649 (IQR: 1132–4394 copies/ml) at a median time of 3.0 (IQR 2.7–3.6) years after their baseline undetectable value.

## Discussion

Our finding of an average decline of 3.2 cells/mm^3^ per month or 38.4 cells/mm^3^ per year is lower than that reported for men who have sex with men infected with subtype B HIV; Mellors et al reported annual CD4 declines of 64 cells/mm^3^ per year overall and 36.3 cells/mm^3^ per year among participants with HIV RNA of <500 copies/ml, and 76 cells/mm^3^ per year for those with an HIV RNA >30,000 copies/mL. Recent sero-converters, infected with HIV subtypes A or D from Uganda appeared to have a more rapid CD4 decline than what we report, and subtype D infected individuals declined more rapidly than subtype C. However, our results are similar to those from a clinical cohort from South Africa in which the CD4 decline among those with a CD4 at clinic entry of 201–350 cells/mm^3^ was 20.5 and for those with >500 cells/mm^3^ at clinic entry was 47.1 [6]; the equivalent results in our group were declines of 17.2 and 48.5 cells/mm^3^ per annum, respectively. We posit that differences in CD4 declines reflect subtype differences, differences between recent sero-converters and chronically infected individuals, or innate host susceptibility to HIV infection. Interestingly, an episode TB was associated with the largest decline in CD4. Although we did not confirm infection with subtype C in this study, HIV subtype C is particularly important, as it is the most common subtype in South Africa and globally.

We included relatively healthy HIV-infected individuals as opposed to those entering care when ill. Subjects entering follow-up due to illness may not provide an accurate estimate of CD4 decline because CD4 fluctuates around the time of an acute illness, and the potential for a more rapid CD4 decline. In addition, the excellent follow-up in the parent study reduces potential bias due to early loss of participants with the steepest CD4 declines.

We observed that 2.6% of our cohort had the long term non-progressor (LTNP) phenotype as their CD4 count CD4 remained >500 cell/mm^3^ for at least five years) and 3.1% of the cohort were found to be elite controllers (ECs) with repeated HIV RNA measures of <400 copies/mL despite not receiving ART. These unusual phenotypes may provide clues to vaccine development and novel antiretroviral therapies However, our inclusion criteria probably resulted in higher rates of both rare phenotypes than in the general population of HIV-infected adults as we excluded those with advanced HIV disease.

This study is an important addition to understanding CD4 decline in HIV-infected adults from a sub-Saharan African country with a massive HIV burden. It includes predominantly healthy individuals with low mortality whose CD4 counts were measured at scheduled visits in contrast to other cohorts with an over-representation of ill individuals for whom CD4 count may be declining due to opportunistic illnesses or infections. The study also provides important data that will likely not be collectable once South Africa follows international trends [12] and raises the CD4 count threshold for ART initiation to 500 cells/mm^3^. Moreover, our data clearly shows the important prognostic role of a viral load estimates at entry into care, supporting calls for consideration for the inclusion of viral load estimates at HIV diagnosis to identify those likely to be efficient transmitters of HIV [13], [14].
