# Correction: CD4 and Viral Load Dynamics in Antiretroviral-Naïve HIV-Infected Adults from Soweto, South Africa: A Prospective Cohort

**DOI:** 10.1371/journal.pone.0130509

**Published:** 2015-06-10

**Authors:** Neil A. Martinson, Nikhil Gupte, Reginah Msandiwa, Lawrence H. Moulton, Grace L. Barnes, Malathi Ram, Glenda Gray, Chris Hoffmann, Richard E. Chaisson

There is an error in the fourth sentence of the Results section in the Abstract. The correct sentence is: The overall mean decline in CD4 count was 3.2 cells/mm^3^ per annum.
