# Correction: Correction: CD4 and Viral Load Dynamics in Antiretroviral-Naïve HIV-Infected Adults from Soweto, South Africa: A Prospective Cohort

**DOI:** 10.1371/journal.pone.0133093

**Published:** 2015-07-13

**Authors:** The PLOS ONE Staff

There is an error in the fourth sentence of the Results section of the Abstract in the correction published on June 10, 2015. The correct sentence is: The overall mean decline in CD4 count was 3.2 cells/mm^3^ per month. The publisher apologizes for the error.
